# Targeting RNA transcription and translation in ovarian cancer cells with pharmacological inhibitor CDKI-73

**DOI:** 10.18632/oncotarget.2296

**Published:** 2014-07-31

**Authors:** Frankie Lam, Abdullahi Y. Abbas, Hao Shao, Theodosia Teo, Julian Adams, Peng Li, Tracey D. Bradshaw, Peter M. Fischer, Elisabeth Walsby, Chris Pepper, Yi Chen, Jian Ding, Shudong Wang

**Affiliations:** ^1^ Centre for Drug Discovery and Development, Sansom Institute for Health Research and School of Pharmacy and Medical Sciences, University of South Australia, Adelaide, South Australia, Australia; ^2^ School of Pharmacy and Centre for Biomolecular Sciences, University of Nottingham, University Park, Nottingham, United Kingdom; ^3^ Cardiff CLL Research Group, Institute of Cancer and Genetics, School of Medicine, Cardiff University, Health Park, Cardiff, United Kingdom; ^4^ Shanghai Institute of Materia Medica, Chinese Academy of Sciences, Shanghai, People's Republic of China

**Keywords:** CDK9, shRNA, Mnks, eIF4E, kinase inhibitors, PI3K/Akt/mTOR, Ras/Raf/MAPK, Flavopiridol, CGP57380, apoptosis, transcription, translation, drug development

## Abstract

Dysregulation of cellular transcription and translation is a fundamental hallmark of cancer. As CDK9 and Mnks play pivotal roles in the regulation of RNA transcription and protein synthesis, respectively, they are important targets for drug development. We herein report the cellular mechanism of a novel CDK9 inhibitor CDKI-73 in an ovarian cancer cell line (A2780). We also used shRNA-mediated CDK9 knockdown to investigate the importance of CDK9 in the maintenance of A2780 cells. This study revealed that CDKI-73 rapidly inhibited cellular CDK9 kinase activity and down-regulated the RNAPII phosphorylation. This subsequently caused a decrease in the eIF4E phosphorylation by blocking Mnk1 kinase activity. Consistently, CDK9 shRNA was also found to down-regulate the Mnk1 expression. Both CDKI-73 and CDK9 shRNA decreased anti-apoptotic proteins Mcl-1 and Bcl-2 and induced apoptosis. The study confirmed that CDK9 is required for cell survival and that ovarian cancer may be susceptible to CDK9 inhibition strategy. The data also implied a role of CDK9 in eIF4E-mediated translational control, suggesting that CDK9 may have important implication in the Mnk-eIF4E axis, the key determinants of PI3K/Akt/mTOR- and Ras/Raf/MAPK-mediated tumorigenic activity. As such, CDK9 inhibitor drug candidate CDKI-73 should have a major impact on these pathways in human cancers.

## INTRODUCTION

Cyclin dependent kinases (CDKs) are a family of serine/threonine kinases, whose activity is tightly associated with specific cyclin co-factors. Over the last decade, more than 20 CDKs have been characterized and are generally classified into two major groups, based on whether their primary role is involved in the control of cell cycle progression or the regulation of transcription. Multiple CDKs control the cell cycle and are essential for normal proliferation, development and homeostasis. CDK4/cyclin D, CDK6/cyclin D and CDK2/cyclin E facilitate the G_1_-S phase transition by sequentially phosphorylating the retinoblastoma protein (pRb), while CDK1/cyclin A, CDK2/cyclin A and CDK1/cyclin B are essential for S-phase progression and G_2_-M transition, respectively [1].

Inhibition of transcriptional CDKs as an effective anti-cancer strategy has gained considerable attention following the observation that many types of cancer cells rely on the production of short-lived mitotic regulatory kinases and apoptosis regulators such as Mcl-1 for their survival [2, 3]. CDK7/cyclin H is a component of transcription factor IIH (TFIIH) that phosphorylates the serine-5 residues within the heptad repeats of RNA polymerase II (RNAPII) C-terminal domain (CTD) to initiate transcription [4, 5]. CDK9/cyclin T, the catalytic subunit of positive transcription elongation factor P-TEFb [6, 7], phosphorylates two elongation repressors, i.e. the DRB-sensitive-inducing factor (DSIF) and the negative elongation factor (NELF), and serine-2 of the CTD heptad repeats to facilitate a productive transcription elongation [3, 8]. Apart from having a role in RNA transcription, CDK7 is also a CDK-activating kinase (CAK), which phosphorylates and activates multiple cell cycle CDKs [5]. In contrast, CDK9 appears to have a minimal effect on cell cycle regulation [9].

Over the last decade, an intensive search for pharmacological CDK inhibitors has led to the development of several clinical candidates and to the realization that inhibition of the transcriptional CDKs underlies their anti-tumor activity [3, 10]. Flavopiridol (alvocidib), the first CDK inhibitor to enter clinical trials, is the most potent CDK9 inhibitor identified to date and has demonstrated marked anti-tumor activity in chronic lymphocytic leukemia (CLL) [11, 12]. Flavopiridol has been shown to inhibit multiple CDKs [13] and other kinases [14], but the primary mechanism responsible for its observed anti-tumor activity in CLL appeared to be the CDK9-mediated down-regulation of transcription of anti-apoptotic proteins [15].

R-Roscovitine (seliciclib) is the first orally bioavailable CDK inhibitor that targets CDK2, CDK7 and CDK9 (IC_50_ = 0.1, 0.5 and 0.8 μM, respectively) [16-18]. Initial evaluation in clinical trials indicated its well tolerance and some evidence of disease stabilization was reported [10]. Phase-II clinical trials are currently underway in patients with non-small-cell lung carcinoma. R-Roscovitine has demonstrated selective induction of apoptosis in cancer cells by down-regulation of anti-apoptotic proteins through transcriptional CDK inhibition [19, 20]. Other CDK inhibitors, including AZD5438 [21], R547 [22, 23] and AT519 [24], have also been evaluating in clinical trials.

Recently, the first-in-class CDK4/6 inhibitor PD 0332991 [25, 26] (palbociclib) has demonstrated marked anti-tumor activity in post-menopausal women with estrogen-receptor positive and HER2-negative advanced breast cancer. However, non-luminal/basal subtypes were resistant to PD 0332991. Cell cycle analysis showed G_0_/G_1_ arrest in sensitive cell lines and Western blot analysis demonstrated that retinoblastoma phosphorylation (pRb) is blocked in sensitive, but not resistant lines. Hyperphosphorylation of Rb is mediated by CDK4 and CDK6 in early G_1_ phase through the interaction with cyclin D. This results in Rb inactivation and release of transcription factors that allows cells progress toward S phase. PD 0332991 showed significant hematologic toxicity and dose reduction for hematologic toxicity was required for 24% of patients in clinical trials [27].

Mitogen-activated protein kinase (MAPK)-interacting kinases (Mnks) are also members of the serine/threonine kinase family, and are emerging anti-cancer targets [28]. As one of the key effectors for the MAPK pathways, Mnks are activated by the extracellular signal-related kinase (Erk) and p38 kinase in response to extracellular stimuli. Subsequently, Mnks phosphorylate residue serine-209 of the eukaryotic initiation factor 4E (eIF4E), a key component of translational control [29]. Recent findings suggested that eIF4E is a key determinant of PI3K/Akt/mTOR- and Ras/Raf/MAPK-mediated tumorigenic activity, and that targeting eIF4E should have a major impact on these pathways in human cancers [29]. Phosphorylation of eIF4E by Mnks at serine-209 is considered to be critical for the oncogenic role of eIF4E. It has demonstrated that mice carrying a serine-209Ala eIF4E mutant were incapable of developing cancer. While Mnk1/2-double knock-out tPTEN^−/−^ mice showed diminished tumour growth compared to the parental tPTEN^−/−^ mice. Significantly, the kinase activity of Mnks seemed not to be essential for normal growth [30, 31]. As such, Mnks have emerged as potential effective and low toxicity targets for cancer therapy.

We have previously described a novel CDK9 inhibitor, CDKI-73, as a targeted therapeutic agent for the treatment CLL [32]. CDKI-73 is highly cytotoxic to primary leukemia cells derived from CLL patients (mean LD_50_ = 0.08 μM) and showed >500-fold selectivity for primary leukemia cells over normal B-lymphocytes (LD_50_ = 40.5 μM). Significantly, CDKI-73 retained efficacy in primary CLL samples derived from poor prognostic subsets including those who had p53 mutation/deletion and those who had relapsed following fludarabine-based regimens. The synergistic effect of CDKI-73 with fludarabine was established at a molar ratio of 1:100, and the synergy mechanism was associated with CDKI-73- mediated transcriptional inhibition of Mcl-1, Bcl-2 and XIAP. The study has provided compelling evidence that CDKI-73 represents a promising therapeutic strategy as a single-target agent as well as combination therapeutics with fludarabine for the treatment of CLL and fludarabine-relapsed diseases.

In the present study, we further evaluated the effects of CDKI-73 on human ovarian cancer cells. Ovarian cancer is the second most common gynecologic cancer; new and more effective treatments are urgently required. Screening against a panel of 24 human cancer cell lines showed that CDKI-73 was most cytotoxic against A2780 ovarian cancer cells. We herein report that CDKI-73 targets both CDK9- and eIF4E-related pathways and it may serve as an effective therapeutic agent for the treatment of ovarian cancer, as well as a range of other cancers.

## RESULTS

### CDKI-73 exhibits broad spectrum anti-cancer activity

CDKI-73, a heterocyclic 3-(5-fluoro-4-(4-methyl-2-(methylamino)thiazol-5-yl)pyrimidin-2-ylamino)benzenesulfonamide, was rationally designed and optimized using CDK9 protein structure-guided approach [33]. X-ray crystallographic analysis confirmed that CDKI-73 analogs targeted CDK9 in an ATP-dependent manner [34]. CDKI-73 is one of the most potent CDK9 inhibitors identified to date [32, 33]. However, it also inhibits CDK1, CDK2 and CDK7 in the low nM range in biochemical kinase assays.

We firstly assessed the effects of CDKI-73 on cell viability against a panel of 24 human solid tumor and hematological cancer cell lines using MTT and resazurin assays. The relevant half-maximal inhibitory concentration (IC_50_) values are summarized in Table 1. CDKI-73 has exhibited potent anti-proliferative activities against all tumor cell lines tested with IC_50_ values ranging from 0.007 to 0.573 μM following a 72 h of exposure. CDKI-73 was most potent in A2780 ovarian cancer cells with an IC_50_ = 0.007 μM.

**Table 1:** Effect of CDKI-73 on cell viability by 72 h MTT assay

Human cell line		*IC_50_ (μmol/L) ± SD
Ovarian carcinoma	A2780	0.007 ± 0.001
Epithelial carcinoma	A431	0.057 ± 0.014
Colorectal carcinoma	COLO 205	0.071 ± 0.013
	HCT-116	0.017 ± 0.007
Cervical carcinoma	HeLa	0.031 ± 0.004
Prostate adenocarcinoma	LNCaP	0.057 ± 0.016
	DU-145	0.042 ± 0.005
Breast adenocarcinoma	MCF-7	0.066 ± 0.011
	MDA-MB-231	0.058 ± 0.004
	T47D	0.071 ± 0.002
Pancreatic carcinoma	PANC-1	0.573 ± 0.027
Leukaemia cell line†	HL-60	0.033 ± 0.013
	K562	0.517 ± 0.092
	KG-1	0.045 ± 0.005
	KYO-1	0.467 ± 0.011
	Kasumi-1	0.038 ± 0.001
	ME-1	0.363 ± 0.026
	MOLM-13	0.033 ± 0.010
	MV4-11	0.034 ± 0.005
	NB4	0.054 ± 0.004
	PL-21	0.037 ± 0.003
	SET-2	0.477 ± 0.068
	THP-1	0.062 ± 0.002
	U937	0.012 ± 0.007

* The data given are the mean ± SD derived from at least three replicates.

† Determined using Resazurin assay.

We next carried out time-course proliferation assays with CDKI-73 in A2780 cells compared with that of flavopiridol (Table 2). CDKI-73 inhibited the growth of tumor cells at early time-points, i.e. 24 h, with an IC_50_ value of 0.033 μM; similar to flavopiridol (IC_50_ = 0.049 μM). Prolonged exposure to CDKI-73 (i.e. 72 h) caused a significant reduced cell viability; showing a >5-fold higher potency than flavopiridol. CGP57380, a known Mnk inhibitor [28, 29], was included in the study as a comparator agent with targeted effects on the Mnk-eIF4E pathway. CGP57380 was significantly less active in inhibiting A2780 tumor growth (IC_50_ ≥5 μM) than CDKI-73.

**Table 2:** Effects of CDKI-73, Flavopiridol and CGP57380 on cell viability by MTT time-course experiments

		IC_50_* (μmol/L) ± SD		
Cell line	Compound	24 h	48 h	72 h
A2780	CDKI-73	0.033 ± 0.002	0.010 ± 0.001	0.007 ± 0.001
	Flavopiridol	0.049 ± 0.002	0.056 ± 0.010	0.036 ± 0.003
	CGP57380	9.022 ± 1.068	7.427 ± 1.157	4.935 ± 0.229
A2780 (CDK9 shRNA)	CDKI-73	>10	0.985 ± 0.132	0.386 ± 0.036
	Flavopiridol	>10	0.245 ± 0.113	0.061 ± 0.014

* The data given are the mean ± SD derived from at least three replicates.

### CDK9 shRNA confirms the target specificity of CDKI-73

**Figure 1:**
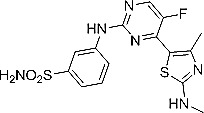
Chemical structure of CDKI-73

To investigate the importance of CDK9 expression in A2780 cells and to assess the target specificity of CDKI-73, we silenced CDK9 expression using a lentivirus-mediated short hairpin RNA (shRNA) strategy [32]. A stable knockdown of CDK9 was achieved to ~90% when compared to non-genetic modified A2780 cells in mRNA transcription (Figure 1A) and protein expression (Figure 1B). These results confirmed the successful knockdown of CDK9 in A2780 cells.

CDK9 knockdown (CDK9KD) A2780 cells have demonstrated significant resistance to CDKI-73. After treatment of CDK9KD cells with CDKI-73 for a period of 24 h, no cytotoxic effect was observed (IC_50_ >10 μM). Following treatment for 48 h and 72 h, CDKI-73 caused cell death, but revealed >98- and >50-fold reduced potency, respectively, compared to CDKI-73 in A2780 parental cells (Table 2), confirming CDK9 targeting specificity of CDKI-73. CDK9KD A2780 cells also showed reduced sensitivity towards flavopiridol at 24 h treatment period, but the cells regained sensitivity by 48 and 72 hours, suggesting off-target effects of flavopiridol [13].

**Figure 2:**
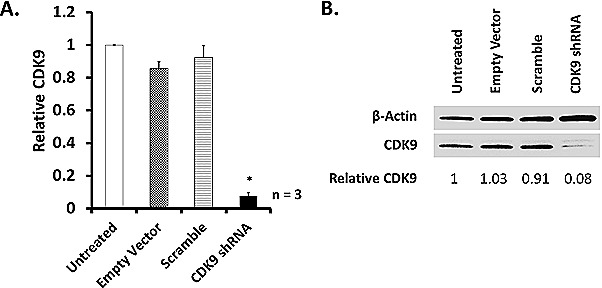
ShRNA-mediated CDK9 knockdown in A2780 cells Lentivirus-containing CDK9 shRNA were used to transfect A2780 cells as previously described [32]. The level of CDK9 knockdown was quantified with three independent verifications by (A) Real Time-quantitative PCR (RT-qPCR) and (B) Western blot. (*) indicates p ≤ 0.05 compared to A2780 untransfected cells.

### CDKI-73 activates caspase-3/7 and induces apoptosis

**Figure 3:**
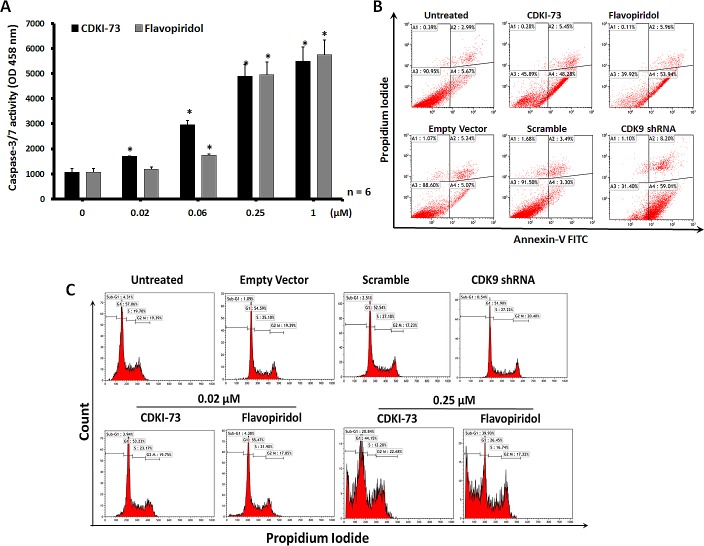
Induction of apoptosis by CDKI-73 and CDK9 shRNA (A) Caspase-3/7 activity in A2780 ovarian cancer cells following treatment with CDKI-73 or flavopiridol for 24 h. Dose-dependent activation of caspase-3/7 was observed in A2780 cells starting from 0.02 μM CDKI-73 and 0.06 μM flavopiridol. Vertical bars represent the mean ± SD of three independent experiments. Values significantly different from the untreated samples are marked with an asterisk (*) (p ≤ 0.05). (B) shRNA-mediated CDK9 knockdown and treatment with 0.25 μM CDKI-73 or flavopiridol induced apoptosis in A2780 cells following treatment for 48 h as analyzed by annexin-V/PI assay. (C) Cell cycle analysis of A2780 cells after treatment with CDKI-73 or flavopiridol for 24 h. No detectable cell cycle effect was observed with CDKI-73- or flavopiridol-treated cells at 0.02 μM, the concentration causing a significant caspase-3/7 activation; at a higher concentration, *i.e.* 0.25 μM CDKI-73 or flavopiridol induced the significant numbers of sub-G_1_ cells, indicating cell death. The shRNA CDK9KD cells also showed no cell cycle disturbance.

To investigate whether apoptosis was the relevant mode of action observed in the viability assay, we measured the activity of caspase-3/7 in A2780 cells after exposure to CDKI-73 or flavopiridol for a period of 24 h. As shown in Figure 3A, 0.02 μM CDKI-73 significantly increased caspase-3/7 activity in A2780 cells and in a dose-dependent manner. Induction of the same level of caspase-3/7 requires 0.06 μM flavopiridol, consistent with its lower potency compared to CDKI-73 in the cytotoxicity assay.

Induction of apoptosis in A2780 cells by either shRNA-mediated CDK9 knockdown or pharmacological inhibitors was further confirmed by annexin-V/propidium iodide (PI) dual staining following treatment of cells for a period of 48 h (Figure 3B). ShRNA-mediated CDK9 inhibition caused 59% apoptosis, as indicated by the annexin-V positive and PI negative (annexin-V+/PI-) cells. Similarly, treatment of A2780 cells with CDKI-73 or flavopiridol at 0.25 μM resulted in > 48% increase in annexin-V+/PI- cells when compared to untreated cells. Taken together, these results confirmed that A2780 cells are dependent on CDK9 activity for survival and inhibition of CDK9 activity by either genetic knockdown or pharmacological inhibitor promotes cellular apoptosis.

As CDKI-73 was shown to inhibit CDK1 and CDK2 by *in vitro* kinase assays [32], we investigated the cell cycle effect of CDKI-73 on A2780 cells compared to that of CDK9KD cells. As shown in Figure 3C, no significant difference in the cell cycle profiles was observed in CDK9KD A2780 cells compared to the transfection controls (i.e. empty vector and scramble) and untransfected cells, confirming a lack of influence of CDK9 on cell cycle. Similarly, no cell cycle effect was observed with A2780 cells after exposure to 0.02 μM CDKI-73 for 24 h, despite the fact that the same conditions have given rise to a significant caspase-3/7 activity in the cells (Figure 3A). At a higher concentration, i.e. 0.25 μM, CDKI-73 induced substantial sub-G_1_ events, an indicative of cell death. Flavopiridol showed similar cell cycle profiles to CDKI-73.

### CDKI-73 down-regulates the phosphorylation of RNAPII and eIF4E

**Figure 4:**
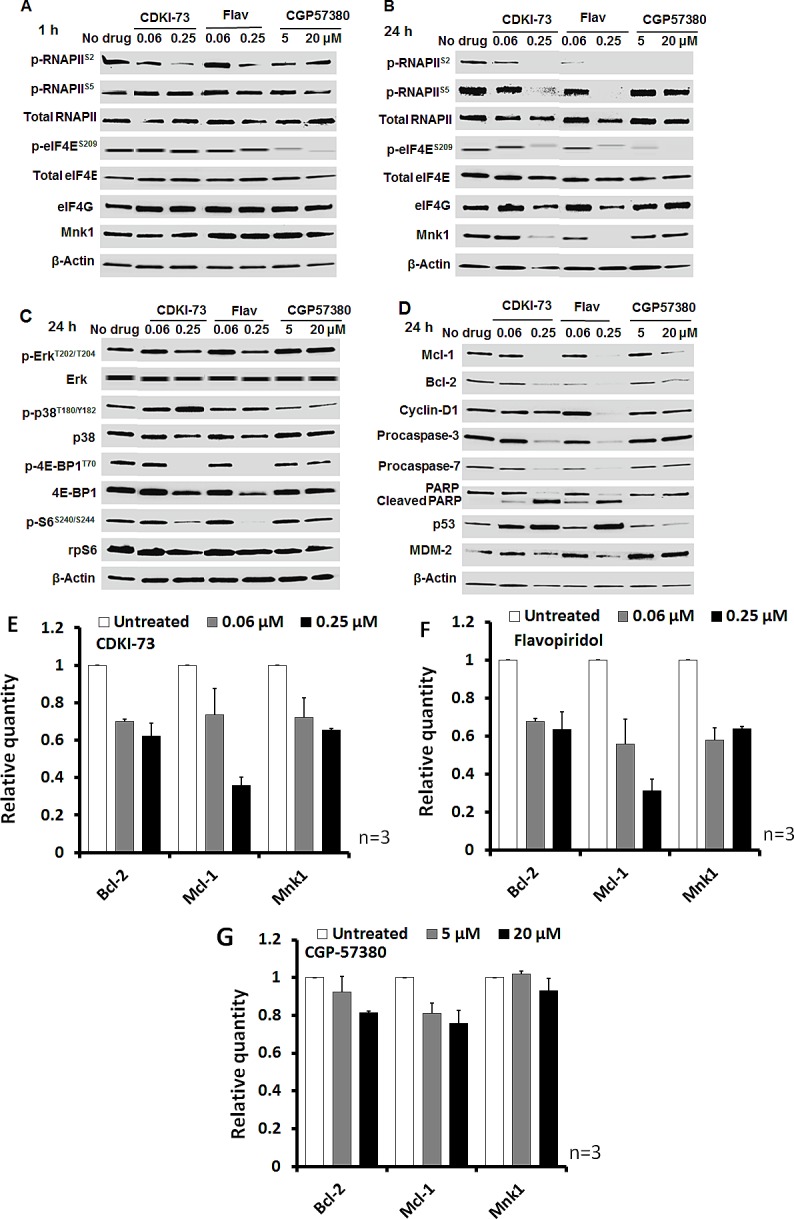
Mechanistic investigation of the molecular effects by Western blotting and RT-qPCR analysis (A) A2780 cells were treated with CDKI-73, flavopiridol or CGP57380 for 1 h. CDKI-73 and flavopiridol reduced phosphorylation of RNAPII at serine-2 at 0.06 and 0.25 μM, respectively, while CGP57380 only affected the phosphorylation of eIF4E (p-eIF4E^S209^) via inhibition of Mnk1. (B) After 24 h drug exposure, CDKI-73 or flavopiridol also abolished p-eIF4E^S209^ at 0.25 μM, indicating cellular inhibition of Mnk kinase activity. The same treatment with CDKI-73 or flavopiridol caused a loss in Mnk1 protein expression. CGP57380-treated cells abrogated p-RNAPII^S2^ as well as p-eIF4E^S209^ with a minimal effect on Mnk1 protein level. (C) 0.25 μM CDKI-73 or flavopiridol caused little changes in the phosphorylation of Erk and p38 MAPK; however, inhibited the 4E-BP1 phosphorylation (p-4E-BP1^Thr70^) by 24 h. CGP57380 had a minimal effect on these proteins. (D) At 0.25 μM, CDKI-73 or flavopiridol suppressed the expression of anti-apoptotic proteins Mcl-1 and Bcl-2, leading to caspase-3/7 activation and PARP cleavage. In addition, MDM2 protein level was reduced and this was accompanied by the up-regulation of p53. CGP57380 reduced Mcl-1 and Bcl-2 proteins at a concentration of 20 μM, but caused little alterations in caspase-3 activity and PARP cleavage, indicating a minimal apoptotic effect. DMSO diluent was used as a control in each experiment and β-actin was used as an internal loading control. A representative blot is selected from at least two independent repeats of experiments. The expression of mRNA encoding Bcl-2, Mcl-1 and Mnk1 were reduced by (E) CDKI-73 and (F) Flavopiridol in A2780 cells after treatment for 4 h starting at a concentration of 0.06 μM in a dose-dependent manner. (G) CGP57380 showed a minimal effect on these transcripts. Actin mRNA was used as a reference sequence.

We next investigated the effect of CDKI-73 on protein expression using Western blotting. A2780 cells were incubated with CDKI-73 for 1 h. The level of the phosphorylated RNAPII at serine-2 (p-RNAPII^S2^, Figure 4A) was suppressed, starting from 0.06 μM in a dose-dependent manner. In contrast, the level of the phosphorylated serine-5 of CTD RNAPII (p-RNAPII^S5^), and the proteins involved in the Mnk-eIF4E axis were not affected, indicating that CDK9 is the primary target for CDKI-73. Flavopiridol also reduced CDK9 activity, but this was only evident at a higher concentration (i.e. 0.25 μM). CGP57380 demonstrated potent anti-Mnk activity by blockage of eIF4E phosphorylation at serine-209 (p-eIF4E^S209^) at 5μM. This compound had little effect on CDK9 and CDK7 kinase activity following 1 h-treatment.

By extending the treatment to 24 h, both CDKI-73 and flavopiridol abolished phosphorylation at serine-2 and serine-5 of RNAPII at 0.25 μM, indicative of their cellular CDK9 and CDK7 inhibitory activities (Figure 4B). Interestingly, both compounds were capable of blocking the Mnk-mediated eIF4E phosphorylation at the serine-209 at the same concentration. Expectedly, CGP57380 inhibited the level of p-eIF4E^S209^ at 5 μM. However, it was surprising that CGP57380 also caused a loss in the phosphorylation of RNAPII (p-RNAPII^S2^). No changes in the levels of total RNAPII and eIF4E proteins were detected in cells treated with compounds. However, the level of Mnk1 expression was reduced by 0.25 μM CDKI-73 or flavopiridol. These observations suggested that CDKI-73 (or flavopiridol) might also target the proteins involved in the eIF4E-mediated translation in cancer cells.

To assess whether CDKI-73 affected the MAPK and mTOR pathways, we examined their respective upstream protein expression and kinase activities. Mnk1 kinase activity is known to be regulated by p38 MAPK and Erk through phosphorylation at Thr197 and Thr202, respectively [35]. p38 MAPK is activated by MKK3/6 through phosphorylation at its Thr180 and Tyr182 residues, wheras Erk is phosphorylated by MEK1 at Thr202 and Tyr204 residues. Western blotting analysis of A2780 cells following exposure to compounds for 24 h revealed that, as shown in Figure 4C, neither CDKI-73 nor flavopiridol had any effect on the Erk and p38 MAPK pathways; no significant change in the levels of phosphorylated Erk (i.e. p-Erk^T202/ T204^), and p38 MAPK (i.e. p-p38^T180/Y182^) was detected, indicating their Mnk selectivity profile. However, the phosphorylation of eIF4E binding protein (4E-BP1) at Thr70, i.e. p-4E-BP1^T70^, was blocked by 0.25 μM CDKI-73 and flavopiridol (Figure 4C). A reduction of 4E-BP1 protein was also observed. CGP57380 inhibited p38 phosphorylation, but showed a minimal effect on 4E-BP1.

We further examined the changes of anti-apoptotic and onco-proteins including Mcl-1, Bcl-2 and cyclin D1, which are regulated by CDK9- and eIF4E-mediated transcription and translation, respectively [3, 29]. The expression of anti-apoptotic proteins Mcl-1 and Bcl-2 was significantly reduced in A2780 cells after treatment with 0.25 μM CDKI-73 or flavopiridol, as well as 20 μM CGP57380 (Figure 4D). The treatment with CDKI-73 or flavopiridol also suppressed cyclin D1 protein expression. The loss in procaspase-3 and -7, together with the increase in cleaved PARP confirmed the induction of apoptosis by CDKI-73. Being consistent with previous studies of CDK9 inhibitors [13, 36], CDKI-73 increased p53 protein expression concomitantly with down-regulation of MDM2, a short-lived protein responsible for p53 degradation. Taken together, CDKI-73 demonstrated potent CDK9 inhibition as well as, the ability to block the eIF4E pathway by suppressing Mnk and 4E-BP1 activities. This resulted in the reduction of Mcl-1 and Bcl-2 and promoted caspase-3/7-dependent apoptosis in A2780 cells.

### CDK9 inhibition causes the down-regulation of Mnk1

**Figure 5:**
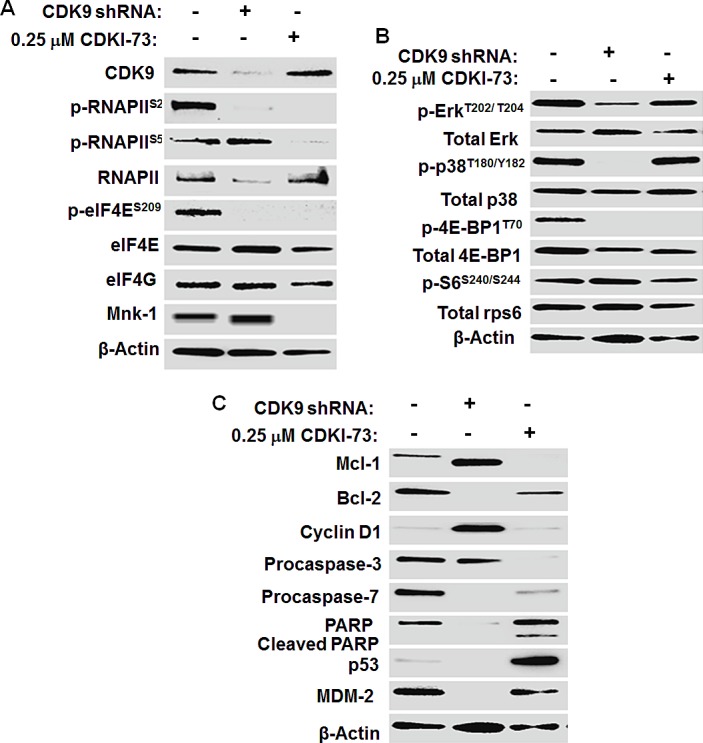
Mechanism of action of CDKI-73 in A2780 cells compared to shRNA-mediated CDK9KD in A2780 cells (A) Western blotting analysis showed the reduced levels of CDK9 protein and p-RNAPII^S2^ in CDK9 shRNA KD A2780 cells. Down-regulation of p-eIF4E^S209^ by shRNA-mediated CDK9KD indicated Mnk1 inhibition. Like CDK9 shRNA, CDKI-73 caused the loss of p-RNAPII^S2^ and p-eIF4E^S209^ in A2780 cells. Unlike CDK9 shRNA, CDKI-73-treated cells showed a reduced p-RNAPII^S5^, indicating CDK7 inhibition at 0.25 μM. (B) Knockdown of CDK9 reduced the phosphorylation of Erk, p38 and 4E-BP1, suggesting a role of CDK9 in MAPK cascade and mTOR-eIF4E pathway. CDKI-73 had little effects on Erk and p38 MAPK and their phosphorylated forms. Like CDK9 shRNA, however, CDKI-73 inhibited the phosphorylation of 4E-BP1. (C) Knockdown of CDK9 suppressed Bcl-2, Mcl-1 (~ 40 kDa), procaspase-3/7 and PARP. CDKI-73-treated cells decreased Mcl-1, Bcl-2, procaspase-3/7 protein levels and induced cleaved PARP. DMSO diluent was used as a control in each experiment and β-actin was used as an internal loading control. A representative blot is selected from at least two independent repeats of experiments.

To determine whether there is any inter-dependence between mRNA transcription and eIF4E translation, we examined the transcription of Mnk1, as well as Bcl-2 and Mcl-1 in A2780 cells. After 4 h treatment with CDKI-73 (Figure 4E) or flavopiridol (Figure 4F), the mRNA levels of all three genes were reduced in a dose-dependent manner. In contrast, CGP57380 showed a minimal effect on these transcripts (Figure 4G), suggesting the CDK9-mediated transcriptional inhibition of Mnk1, Mcl-1 and Bcl-2. The effects on CDK9 and Mnk-eIF4E pathways were further examined in the CDK9KD A2780 cells in comparison with CDKI-73-treated A2780 cells. As expected, down-regulation of CDK9 protein, its kinase activity (i.e. p-RNAPII^S2^), and RNAPII was observed in the CDK9KD cells (Figure 5A). Significantly, shRNA-mediated CDK9 inhibition also caused down-regulation of Mnk1 expression, resulting in a loss of eIF4E phosphorylation at serine-209, consistent with the CDK9-Mnk targeting mechanism of CDKI-73. Furthermore, the CDK9 knockdown caused loss in the levels of phosphorylated Erk, p38 and 4E-BP1 (Figure 5B). A slight reduction in p38 and 4E-BP1 protein expression was also observed. In contrast, other than the abrogated phosphorylation of 4E-BP1, CDKI-73 had little effect on the levels of other proteins examined. Ribosomal protein S6 (rpS6) and its phosphorylation (i.e. p-S6^S240/S244^) were not affected by either shRNA-mediated CDK9KD or CDKI-73. Induction of apoptosis by CDK9 shRNA was also associated with the loss of Mcl-1 and Bcl-2, the induction of caspase-3/7 activity and PARP cleavage.

## DISCUSSION

Dysregulations of mRNA transcription and protein synthesis are common events in human cancers. CDK9 and eIF4E are key regulators in mRNA transcriptional elongation and translational initiation, respectively. CDK9 inhibition as an effective anti-cancer strategy has gained strong support in recognizing that cancer cells rely on the production of short-lived apoptosis regulators and mitotic regulatory kinases for survival [2, 3]. Our own studies have also demonstrated that CDK9 inhibitors can achieve anti-cancer activity with minimal toxicity to non-transformed cells [13, 32]. Control of mRNA translation plays a critical role in cell growth, proliferation and differentiation. Most mRNAs are translated in a cap-dependent manner. A key player in the regulation of translation is the mRNA cap-binding protein eIF4E, the rate-limiting member of the eIF4F complex. Overexpression of eIF4E has been found in a range of human cancers and phosphorylation of eIF4E by Mnks is responsible for the oncogenic role of eIF4E [28, 30]. Inhibition of Mnk-mediated eIF4E activity is believed to have a major impact on the PI3K/Akt/mTOR and Ras/Raf/MAPK pathways in human cancers [29, 37].

CDKI-73 is one of the most potent CDK9 inhibitors identified to date, and has been shown to induce apoptosis in CLL patient samples with minimal toxicity [32]. CGP57380 is a potent Mnk1 inhibitor (*K*_i_ = 0.34 μM) [38], and has served as a tool compound to reveal the role of eIF4E in cell biology [39]. CGP57380 also inhibits other kinases including CK1 (with a similar potency to Mnk1), BRSK2, MKK1 and Pim3 [40], however it dose not target CDKs. CGP57380 has been shown to block eIF4E phosphorylation in HCT-116 and B16 cell lines [41]. To determine whether targeting the Mnk-eIF4E axis in cancer cells is an additional mechanism to CDK9 inhibition or a consequence of CDK9 pathway alteration, we conducted experiments utilizing shRNA-mediated CDK9 inhibition to compare with the pharmacological effects of CDKI-73. Flavopiridol and CGP57380 were used as comparators for assessments of CDK9 and Mnk1 pharmacological mechanism, respectively.

Despite the fact that CDKI-73 was highly cytotoxic to a wide range of tumor cell lines, particularly to A2780 ovarian cancer cells (IC_50_ = 0.007 μM, Table 1), it had a reduced potency in the CDK9KD A2780 cells (IC_50_ = 0.386 – < 10 μM, Table 2). These confirmed the CDK9-targeting specificity of CDKI-73, as the down-regulation of anti-apoptotic proteins in the CDK9 knockdown cells reduced the sensitivity towards CDK9 inhibitor [42]. CDKI-73 induced significant caspase-3/7 activity at 0.02 μM (Figure 3A), and the concentration caused no cell cycle effect (Figure 2A and 2C), confirming its CDK9 inhibition mediated apoptosis. CDKI-73 induced substantial apoptotic populations; resulting in ~48% apoptotic cells as detected in annexin-V assay (Figure 3B). Cell cycle analysis confirmed that CDKI-73 caused substantial cell death (i.e. sub-G_1_, Figure 3C).

In line with our previous findings [32, 33], CDKI-73 inhibits cellular CDK9-mediated RNAPII transcription. Western blotting of A2780 cells after exposure to 0.06 μM CDKI-73 for 1 h reduced the phosphorylation CTD RNAPII at serine-2 (Figure 4A). In addition to targeting CDK9; and CDK7 to a lesser extent, CDKI-73 has shown to decrease the phosphorylation of eIF4E. As shown in Figure 5A, the combined suppression of p-RNAPII^S2^ and p-eIF4E^S209^ were observed in the CDK9 shRNA KD cells, consistent with those shown in CDKI-73-treated cells and suggesting a role of CDK9 in the Mnk-eIF4E axis. The reduced mRNA transcript for Mnk1 as in Figure 5E further confirm that CDKI-73 inhibit Mnk-eIF4E axis through CDK9 transcriptional inhibition. Intriguingly, CGP57380 was shown to suppress the phosphorylation of RNAPII at serine-2. CGP57380 is known to target CK1 with an IC_50_ = 0.51 μM in a biochemical assay [40]. It has been shown that CK1 activity is required for activation of heat shock proteins (Hsps) 70 and 90 [43], which in turn stabilize CDK9 binding to cyclin T1 leading to productive mRNA transcription [44]. Therefore, it is likely that the suppression of p-RNAPII^S2^ by CGP57380 may through its off-target CK1-Hsp inhibitory mechanism.

To further assess whether down-regulation of CDK9 by shRNA affected Mnk up-steam activating kinases, we examined Erk and p38 MAPK phosphorylation in CDK9KD cells, both were abrogated. In contrast, CDKI-73 (or flavopiridol) caused little changes in these proteins (Figures 4C and 5B). It is not understood why CDK9 knockdown should cause the de-phosphorylation of Erk and p38; nevertheless suggesting a role of RNAPII in transcriptional regulation of Erk and p38 upstream modules. It is worth noting that the shRNA-mediated CDK9 inhibition silences the gene expression of CDK9 and hence shutting off the protein synthesis in the cells, whereas pharmacological inhibitor CDKI-73 blocked only the kinase activity of CDK9 without affecting the protein expression. The difference has important implications; for instance, CDK9 knockdown causes alternation not only in a range of mRNA transcripts, but also in the proteins that interact with their binding partners or assemble into macromolecular complexes.

Mcl-1 plays a pivotal role in maintaining mitochondrial outer membrane permeability. CDKI-73 or CDK9 shRNA showed a significant apoptotic effect (Fig 4D and 5C). Reduction in procaspase-3 and procaspase-7 in CDKI-73-treated A2780 cells indicated the conversion of their respective procaspases to the active forms. This was further supported by the increased level of cleaved PARP. Mcl-1 and Bcl-2 were down-regulated following the CDK9 inhibition (Figure 4D, 4E and 5C). Expression of p53 is regulated at the post-translational level by association with its negative regulator MDM2 [45], a short-lived protein known to be reduced by CDK9 inhibition [13]. As expected, CDKI-73 elevated the level of p53 in concordance with a reduced MDM2. Flavopiridol showed similar effects on these proteins. Equally, shRNA-mediated CDK9KD resulted in reduction of procaspase-3, and in abrogation of procaspase-7, PARP and Bcl-2 proteins (Figure 5C).

Interestingly, in concordance with the reduction of Mcl-1 protein (~40 kDa), a smaller species of Mcl-1 (~36 kDa) was detected in the CDK9KD cells. Mcl-1 has been shown to have a triplet of 36-, 38-, and 40-kDa species, and the 36-kDa form is more stable compared to other two larger forms [46]. Structurally, Mcl-1 can be subdivided into the C-terminal region that shares homology with other anti-apoptotic family members, and the N-terminal region which is unstructured. The short half-life of Mcl-1 is thought to reflect the unique features of its N-terminus. The latter contains two sequences rich in proline, glutamic acid, serine and threonine residues (so-called the PEST sequences) that target it for rapid proteasomal degradation [47, 48]. At least three E3 ubiquitin ligases are thought to contribute to this rapid turnover, and the ability of E3 ligases to bind and modify Mcl-1 is dependent on the phosphorylation of Mcl-1 at certain sites within its PEST sequences. Active Erk has been shown to phosphorylate the PEST region which stabilizes Mcl-1. However, the phosphorylation by Erk can also serve as a priming phosphorylation for the subsequent glycogen synthase kinase-3 (GSK3)-mediated phosphorylation, which promotes Mcl-1 ubiquitylation and degradation [49].

Mnks play critical roles in the regulation of protein synthesis and have emerged as anti-cancer targets [28, 29]. Recent studies have shown that the cytotoxic effects of Mnk inhibitors were cell-specific [50] and the anti-proliferative effects of Mnk inhibitors in some solid tumor cells seemed primarily cytostatic, rather than cytotoxic [28]. Despite being a potent Mnk inhibitor, CGP57380 exhibited only modest cytotoxicity in tumor cell lines [28]. We have shown that CGP57380 was capable of reducing the levels of Mcl-1 and Bcl-2 proteins (20 μM, 24 h), but failed to activate caspase-3/7 and induce apoptosis in A2780 cells (Figure 4D). The observations are in agreement with the study of cycloheximide, a general translational inhibitor, which induced Mcl-1 protein decay by inhibiting the translational machinery without affecting cell apoptosis [51]. These suggest that down-regulation of Mcl-1 protein expression may be necessary, but not sufficient for induction of apoptosis, whereas CDK9-targeted transcriptional inhibition of Mcl-1 should be more effective apoptotic strategy in cancer cells.

Over the last decade, selectivity of kinase inhibitors has been the main focus of drug discovery in an attempt to reduce drug side-effects and toxicity. However, it becomes apparent from clinical studies with molecularly targeted therapy that cancers can escape from a given state of oncogene addiction through mutations in alternative pathways because of the frequent genomic instability of cancers. For this reason, as well as tumor heterogeneity, it is unlikely that the use of a single molecular targeted agent will achieve long-lasting remissions or cures in cancers, especially for late-stage disease [52]. Co-targeting key components of oncogenic pathways have recently been proposed as a more effective strategy for developing anti-cancer drugs [29]. With the combined inhibition of CDK9-mediated transcription and Mnk-eIF4E translation, together with its low toxicity in non-transformed cells and favourable pharmacological properties manifested [32], CDKI-73 may offer an opportunity to treat a wide range of human cancers.

## MATERIALS AND METHODS

### Chemical compounds

Synthesis of CDKI-73 has previously been described [33]. Flavopiridol and CGP57380 were purchased from Sigma-Aldrich (Castle Hill, Australia). They were dissolved in dimethylsulfoxide (DMSO) at a stock concentration of 10 mM, and stored at -20°C in small aliquots.

### Cell culture

A2780 cells were purchased from European Collection of Cell Culture (ECACC). All leukemia cell lines were kindly provided by Prof. R. D'Andrea (University of South Australia). All solid tumor cell lines were obtained from the cell bank at the Centre for Drug Discovery and Development, University of South Australia. All cell lines were maintained under 37^o^C, 5% CO_2_, and were cultured in Roswell Park Memorial Institute (RPMI)-1640 with 10% FBS.

### Lentiviral modulation of CDK9 in A2780 cells

Transfection of cells with lentiviral particles containing CDK9 shRNA and verification of the percentage of knockdown were performed as previously reported [32]. Briefly, bacterial glycerol stocks containing the lentiviral plasmid vector pLKO.1-puro with shRNA inserts against CDK9, an empty vector or a scrambled shRNA control were obtained from Sigma Aldrich (Poole, UK). Lentivirus transfected 293T cells were incubated at 37^o^C for 48h before the resulting lentiviral particles were harvested by centrifugation and concentrated using the Clontech Lenti-X concentrator kit (Lonza, Wokingham, UK). Concentrated virus was then added to A2780 cells and incubated for 48h. Lentivirus-transduced cells were then selected by addition of puromycin (1 μg/mL) to the culture for two weeks. The relative expression of CDK9 was subsequently assessed by RT-qPCR and Western blotting.

### Cell viability assay

MTT (3-(4,5-dimethylthiazol-2-yl)-2,5-diphenyltetrazolium bromide) assays were performed in all solid tumor cell lines as reported previously [53]. Compound concentrations required to induce 50 % of cell viability (IC_50_) were calculated using non-linear regression analysis. Resazurin (Sigma Aldrich) assays were performed in all leukemia cell lines as previously described [38].

### Caspase-3/7 assay

Activity of caspase-3/7 was measured using the Apo-ONE Homogeneous Caspase-3/7 kit (Promega, Madison, WI, USA) according to manufacturer instruction and analyzed using EnVision multi-label plate reader (PerkinElmer, Beaconsfield, UK).

### Cell cycle and detection of apoptosis

A2780 (8 × 10^4^) cells were seeded and incubated overnight at 37^o^C, 5% CO_2_. Followed by the treatment with compounds, cells were trypsinized and centrifuged (300 g, 5 min). Cell pellets were collected and fixed with 70% ethanol on ice for 15 min, followed by centrifugation (300 g, 5 min). The collected pellets were incubated with staining solution (50 μg/mL propidium iodide (PI), 0.1 mg/mL RNase A, 0.05% Triton X-100) at 37^o^C for 1 h and analyzed with Gallios flow cytometer (Beckman Coulter, Brea, CA, USA). Apoptosis were assessed with cells collected and processed as described with annexin V-FITC/PI commercial kit (Becton Dickenson). Samples were analyzed with Gallios flow cytometer (Beckman Coulter, Brea, CA, USA) within 1 h of staining. Data were analyzed using Kaluza v1.2 (Beckman Coulter).

### Western blots

Western blotting was performed either with the Simple Western assay by Simon (ProteinSimple, Santa Clara, CA, USA) according to manufacturer instruction [54] or as previously described [13]. Antibodies used were as follows: total RNAPII, phosphorylated RNAPII serine-2 (p-RNAPII^S2^) and serine-5 (p-RNAPII^S5^) (Covance, NJ, USA), 4E-BP1, p-4E-BP1 (pT^70^), β-Actin, caspase-3, caspase-7, CDK9, eIF4E, p-eIF4E (S^209^), eIF4G, p-Erk^T202/Y204^, p-p38^T180/Y182^, p38 MAPK, rpS6, Mcl-1, Mnk1, PARP, cleaved PARP (Cell Signalling Technology, Danvers, MA, USA), Erk (ProteinSimple), MDM-2 (Becton Dickenson, Franklin Lakes, NJ, USA), Bcl-2, cyclin D1, p-S6^S240/S244^, p53 (Dako, Glostrap, Denmark). Both anti-mouse and anti-rabbit immunoglobulin G (IgG) horseradish peroxidase conjugated antibodies were obtained from Dako. Enhanced Chemiluminescence (ECL) reagents were obtained from GE Life Sciences.

### Real Time-Quantitative PCR (RT-qPCR)

RNA extraction was performed using High Pure RNA Isolation Kit (Roche Applied Science, Castle Hill, Australia). 1 μg RNA was used in a 20 μl reverse transcription reaction using Transcriptor First Strand cDNA Synthesis Kit (Roche Applied Science, Castle Hill, Australia). RT-qPCR was carried out in duplicate with cDNA using SYBR Green I dye (Roche Applied Science, Castle Hill, Australia) and performed using LightCycler LC96 (Roche Applied Science, Penzberg, Germany). All primers were purchased from Sigma-Aldrich (Castle Hill, Australia). Relative quantification using E-method established by Roche Applied Science was performed with β-Actin mRNA as reference sequence and untreated samples as study calibrator. cDNA samples were amplified using the following primer pairs with amplification efficiency (E): β-Actin: 5'-ACTCTTCCAGCCTTCCTTC-3' (forward) and 5'-GATGTCCACGTCACACTTC-3'(reverse), E=1.72; Bcl-2:

5'-ATGGGATCGTTGCCTTATGC-3'(forward) and

5'-CAGTCTACTTCCTCTGTGATGTTGT-3' (reverse), E=1.89; CDK9:

5'-AAAACGAGAAGGAGGGGTTCC-3' (forward) and

5'-CCTTGCAGCGGTTATAGGGG-3' (reverse), E=1.92; Mcl-1:

5'-AACAAAGAGGCTGGGATGGG-3' and 5'-TGCCAAACCAGCTCCTACTC-3' (reverse), E=1.80; Mnk1: 5'-AAGGCCATTGAGACACTTCG-3' (forward) and 5'-CCCAAATGAAATAAAGCTCCTG-3' (reverse), E = 1.74.

### Statistical analysis

All experiments with statistics were performed with at least two independent repeats; representative experiments being selected for figures. Statistical significance of differences for experiments was determined using one-way analysis of variance, with a minimal level of significance at *p* ≤0.05.

## References

[1] Lapenna S, Giordano A (2009). Cell cycle kinases as therapeutic targets for cancer. Nat Rev Drug Discov.

[2] Shapiro GI (2006). Cyclin-dependent kinase pathways as targets for cancer treatment. J Clin Oncol.

[3] Wang S, Fischer PM (2008). Cyclin-dependent kinase 9: a key transcriptional regulator and potential drug target in oncology, virology and cardiology. Trends in pharmacological sciences.

[4] Shiekhattar R, Mermelstein F, Fisher RP, Drapkin R, Dynlacht B, Wessling HC, Morgan DO, Reinberg D (1995). Cdk-activating kinase complex is a component of human transcription factor TFIIH. Nature.

[5] Fisher RP (2005). Secrets of a double agent: CDK7 in cell-cycle control and transcription. J Cell Sci.

[6] Price DH (2000). P-TEFb, a cyclin-dependent kinase controlling elongation by RNA polymerase II. Molecular and Cellular Biology.

[7] Garriga J, Grana X (2004). Cellular control of gene expression by T-type cyclin/CDK9 complexes. Gene.

[8] Marshall RM, Grana X (2006). Mechanisms controlling CDK9 activity. Front Biosci.

[9] Garriga J, Bhattacharya S, Calbo J, Marshall RM, Truongcao M, Haines DS, Grana X (2003). CDK9 is constitutively expressed throughout the cell cycle, and its steady-state expression is independent of SKP2. Mol Cell Biol.

[10] Fischer PM, Gianella-Borradori A (2003). CDK inhibitors in clinical development for the treatment of cancer. Expert Opin Investig Drugs.

[11] Byrd JC, Lin TS, Dalton JT, Wu D, Phelps MA, Fischer B, Moran M, Blum KA, Rovin B, Brooker-McEldowney M, Broering S, Schaaf LJ, Johnson AJ, Lucas DM, Heerema NA, Lozanski G (2007). Flavopiridol administered using a pharmacologically derived schedule is associated with marked clinical efficacy in refractory, genetically high-risk chronic lymphocytic leukemia. Blood.

[12] Christian BA, Grever MR, Byrd JC and Lin TS (2009). Flavopiridol in chronic lymphocytic leukemia: a concise review. Clin Lymphoma Myeloma.

[13] Liu X, Shi S, Lam F, Pepper C, Fischer PM, Wang S (2012). CDKI-71, a novel CDK9 inhibitor, is preferentially cytotoxic to cancer cells compared to flavopiridol. International Journal of Cancer.

[14] Caracciolo V, Laurenti G, Romano G, Carnevale V, Cimini AM, Crozier-Fitzgerald C, Gentile Warschauer E, Russo G, Giordano A (2012). Flavopiridol induces phosphorylation of AKT in a human glioblastoma cell line, in contrast to siRNA-mediated silencing of Cdk9: Implications for drug design and development. Cell Cycle.

[15] Chen R, Keating MJ, Gandhi V, Plunkett W (2005). Transcription inhibition by flavopiridol: mechanism of chronic lymphocytic leukemia cell death. Blood.

[16] Meijer L, Raymond E (2003). Roscovitine and Other Purines as Kinase Inhibitors. From Starfish Oocytes to Clinical Trials. Accounts of Chemical Research.

[17] Wang S, McClue SJ, Ferguson JR, Hull JD, Stokes S, Parsons S, Westwood R, Fischer PM (2001). Synthesis and configuration of the cyclin-dependent kinase inhibitor roscovitine and its enantiomer. Tetrahedron: Asymmetry.

[18] McClue SJ, Blake D, Clarke R, Cowan A, Cummings L, Fischer PM, MacKenzie M, Melville J, Stewart K, Wang S, Zhelev N, Zheleva D, Lane DP (2002). In vitro and in vivo antitumor properties of the cyclin dependent kinase inhibitor CYC202 (R-roscovitine). International journal of cancer Journal international du cancer.

[19] Lacrima K, Valentini A, Lambertini C, Taborelli M, Rinaldi A, Zucca E, Catapano C, Cavalli F, Gianella-Borradori A, Maccallum DE, Bertoni F (2005). In vitro activity of cyclin-dependent kinase inhibitor CYC202 (Seliciclib, R-roscovitine) in mantle cell lymphomas. Ann Oncol.

[20] MacCallum DE, Melville J, Frame S, Watt K, Anderson S, Gianella-Borradori A, Lane DP, Green SR (2005). Seliciclib (CYC202, R-Roscovitine) induces cell death in multiple myeloma cells by inhibition of RNA polymerase II-dependent transcription and down-regulation of Mcl-1. Cancer Res.

[21] Anderson M, Andrews DM, Barker AJ, Brassington CA, Breed J, Byth KF, Culshaw JD, Finlay MR, Fisher E, McMiken HH, Green CP, Heaton DW, Nash IA, Newcombe NJ, Oakes SE, Pauptit RA (2008). Imidazoles: SAR and development of a potent class of cyclin-dependent kinase inhibitors. Bioorg Med Chem Lett.

[22] Chu XJ, DePinto W, Bartkovitz D, So SS, Vu BT, Packman K, Lukacs C, Ding Q, Jiang N, Wang K, Goelzer P, Yin X, Smith MA, Higgins BX, Chen Y, Xiang Q (2006). Discovery of [4-Amino-2-(1-methanesulfonylpiperidin-4-ylamino)pyrimidin-5-yl](2,3-diflu oro-6- methoxyphenyl)methanone (R547), a potent and selective cyclin-dependent kinase inhibitor with significant in vivo antitumor activity. J Med Chem.

[23] DePinto W, Chu XJ, Yin X, Smith M, Packman K, Goelzer P, Lovey A, Chen Y, Qian H, Hamid R, Xiang Q, Tovar C, Blain R, Nevins T, Higgins B, Luistro L (2006). In vitro and in vivo activity of R547: a potent and selective cyclin-dependent kinase inhibitor currently in phase I clinical trials. Mol Cancer Ther.

[24] Wyatt PG, Woodhead AJ, Berdini V, Boulstridge JA, Carr MG, Cross DM, Davis DJ, Devine LA, Early TR, Feltell RE, Lewis EJ, McMenamin RL, Navarro EF, O'Brien MA, O'Reilly M, Reule M (2008). Identification of N-(4-piperidinyl)-4-(2,6-dichlorobenzoylamino)-1H-pyrazole-3-carboxamide (AT7519), a novel cyclin dependent kinase inhibitor using fragment-based X-ray crystallography and structure based drug design. J Med Chem.

[25] Fry DW, Harvey PJ, Keller PR, Elliott WL, Meade M, Trachet E, Albassam M, Zheng X, Leopold WR, Pryer NK, Toogood PL (2004). Specific inhibition of cyclin-dependent kinase 4/6 by PD 0332991 and associated antitumor activity in human tumor xenografts. Molecular Cancer Therapeutics.

[26] Finn RS, Dering J, Conklin D, Kalous O, Cohen DJ, Desai AJ, Ginther C, Atefi M, Chen I, Fowst C, Los G, Slamon DJ (2009). PD 0332991, a selective cyclin D kinase 4/6 inhibitor, preferentially inhibits proliferation of luminal estrogen receptor-positive human breast cancer cell lines in vitro. Breast Cancer Res.

[27] Dickson MA, Tap WD, Keohan ML, D'Angelo SP, Gounder MM, Antonescu CR, Landa J, Qin LX, Rathbone DD, Condy MM, Ustoyev Y, Crago AM, Singer S, Schwartz GK (2013). Phase II trial of the CDK4 inhibitor PD0332991 in patients with advanced CDK4-amplified well-differentiated or dedifferentiated liposarcoma. J Clin Oncol.

[28] Diab S, Kumarasiri M, Yu M, Teo T, Proud C, Milne R, Wang S (2014). MAP kinase-interacting kinases-emerging targets against cancer. Chem Biol.

[29] Hou J, Lam F, Proud C, Wang S (2012). Targeting Mnks for cancer therapy. Oncotarget.

[30] Ueda T, Watanabe-Fukunaga R, Fukuyama H, Nagata S, Fukunaga R (2004). Mnk2 and Mnk1 are essential for constitutive and inducible phosphorylation of eukaryotic initiation factor 4E but not for cell growth or development. Mol Cell Biol.

[31] Ueda T, Sasaki M, Elia AJ, Chio II, Hamada K, Fukunaga R, Mak TW (2010). Combined deficiency for MAP kinase-interacting kinase 1 and 2 (Mnk1 and Mnk2) delays tumor development. Proc Natl Acad Sci U S A.

[32] Walsby E, Pratt G, Shao H, Abbas AY, Fischer PM, Bradshaw TD, Brennan P, Fegan C, Wang S and Pepper C (2013). A novel Cdk9 inhibitor preferentially targets tumor cells and synergizes with fludarabine.

[33] Shao H, Shi S, Huang S, Hole AJ, Abbas AY, Baumli S, Liu X, Lam F, Foley DW, Fischer PM, Noble M, Endicott JA, Pepper C, Wang S (2013). Substituted 4-(thiazol-5-yl)-2-(phenylamino)pyrimidines are highly active CDK9 inhibitors: synthesis, X-ray crystal structures, structure-activity relationship, and anticancer activities. Journal of medicinal chemistry.

[34] Hole AJ, Baumli S, Shao H, Shi S, Huang S, Pepper C, Fischer PM, Wang S, Endicott JA, Noble ME (2013). Comparative structural and functional studies of 4-(thiazol-5-yl)-2-(phenylamino)pyrimidine-5-carbonitrile CDK9 inhibitors suggest the basis for isotype selectivity. Journal of medicinal chemistry.

[35] Waskiewicz AJ, Johnson JC, Penn B, Mahalingam M, Kimball SR, Cooper JA (1999). Phosphorylation of the cap-binding protein eukaryotic translation initiation factor 4E by protein kinase Mnk1 in vivo. Mol Cell Biol.

[36] Wang S, Griffiths G, Midgley CA, Barnett AL, Cooper M, Grabarek J, Ingram L, Jackson W, Kontopidis G, McClue SJ, McInnes C, McLachlan J, Meades C, Mezna M, Stuart I, Thomas MP (2010). Discovery and characterization of 2-anilino-4- (thiazol-5-yl)pyrimidine transcriptional CDK inhibitors as anticancer agents. Chemistry & biology.

[37] Hay N (2010). Mnk earmarks eIF4E for cancer therapy. Proc Natl Acad Sci U S A.

[38] Diab S, Teo T, Kumarasiri M, Li P, Yu M, Lam F, Basnet SK, Sykes MJ, Albrecht H, Milne R, Wang S (2014). Discovery of 5-(2-(phenylamino)pyrimidin-4-yl)thiazol-2(3H)-one derivatives as potent Mnk2 inhibitors: synthesis, SAR analysis and biological evaluation. ChemMedChem.

[39] Buxade M, Parra-Palau JL, Proud CG (2008). The Mnks: MAP kinase-interacting kinases (MAP kinase signal-integrating kinases). Front Biosci.

[40] Bain J, Plater L, Elliott M, Shpiro N, Hastie CJ, McLauchlan H, Klevernic I, Arthur JS, Alessi DR, Cohen P (2007). The selectivity of protein kinase inhibitors: a further update. The Biochemical journal.

[41] Konicek BW, Stephens JR, McNulty AM, Robichaud N, Peery RB, Dumstorf CA, Dowless MS, Iversen PW, Parsons S, Ellis KE, McCann DJ, Pelletier J, Furic L, Yingling JM, Stancato LF, Sonenberg N (2011). Therapeutic inhibition of MAP kinase interacting kinase blocks eukaryotic initiation factor 4E phosphorylation and suppresses outgrowth of experimental lung metastases. Cancer Res.

[42] Manohar SM, Rathos MJ, Sonawane V, Rao SV, Joshi KS (2011). Cyclin-dependent kinase inhibitor, P276-00 induces apoptosis in multiple myeloma cells by inhibition of Cdk9-T1 and RNA polymerase II-dependent transcription. Leuk Res.

[43] Muller P, Ruckova E, Halada P, Coates PJ, Hrstka R, Lane DP, Vojtesek B (2013). C-terminal phosphorylation of Hsp70 and Hsp90 regulates alternate binding to co-chaperones CHIP and HOP to determine cellular protein folding/degradation balances. Oncogene.

[44] O'Keeffe B, Fong Y, Chen D, Zhou S, Zhou Q (2000). Requirement for a kinase-specific chaperone pathway in the production of a Cdk9/cyclin T1 heterodimer responsible for P-TEFb-mediated tat stimulation of HIV-1 transcription. J Biol Chem.

[45] Demidenko ZN, Blagosklonny MV (2004). Flavopiridol induces p53 via initial inhibition of Mdm2 and p21 and, independently of p53, sensitizes apoptosis-reluctant cells to tumor necrosis factor. Cancer Res.

[46] Stewart DP, Koss B, Bathina M, Perciavalle RM, Bisanz K, Opferman JT (2010). Ubiquitin-independent degradation of antiapoptotic MCL-1. Mol Cell Biol.

[47] Warr MR, Shore GC (2008). Unique biology of Mcl-1: therapeutic opportunities in cancer. Curr Mol Med.

[48] Quinn BA, Dash R, Azab B, Sarkar S, Das SK, Kumar S, Oyesanya RA, Dasgupta S, Dent P, Grant S, Rahmani M, Curiel DT, Dmitriev I, Hedvat M, Wei J, Wu B (2011). Targeting Mcl-1 for the therapy of cancer. Expert Opin Investig Drugs.

[49] Gores GJ, Kaufmann SH (2012). Selectively targeting Mcl-1 for the treatment of acute myelogenous leukemia and solid tumors. Genes Dev.

[50] Wheater MJ, Johnson PW, Blaydes JP (2010). The role of MNK proteins and eIF4E phosphorylation in breast cancer cell proliferation and survival. Cancer Biol Ther.

[51] Iglesias-Serret D, Pique M, Gil J, Pons G, Lopez JM (2003). Transcriptional and translational control of Mcl-1 during apoptosis. Archives of Biochemistry and Biophysics.

[52] Gottesman MM, Fojo T, Bates SE (2002). Multidrug resistance in cancer: role of ATP-dependent transporters. Nat Rev Cancer.

[53] Wang S, Meades C, Wood G, Osnowski A, Anderson S, Yuill R, Thomas M, Mezna M, Jackson W, Midgley C, Griffiths G, Fleming I, Green S, McNae I, Wu SY, McInnes C (2004). 2-Anilino-4-(thiazol-5-yl)pyrimidine CDK inhibitors: synthesis, SAR analysis, X-ray crystallography, and biological activity. Journal of medicinal chemistry.

[54] Rustandi RR, Loughney JW, Hamm M, Hamm C, Lancaster C, Mach A, Ha S (2012). Qualitative and quantitative evaluation of Simon, a new CE-based automated Western blot system as applied to vaccine development. Electrophoresis.

